# Measuring physiotherapy performance in patients with osteoarthritis of the knee: A prospective study

**DOI:** 10.1186/1472-6963-8-145

**Published:** 2008-07-08

**Authors:** Gro Jamtvedt, Kristin Thuve Dahm, Inger Holm, Signe Flottorp

**Affiliations:** 1 Norwegian Knowledge Centre for the Health Services, P.O. Box 7004, St. Olavs plass, 0103 Oslo, Norway; 2 Centre for Evidence Based Practice, Bergen University College, Bergen, Norway; 3 Section of Health Science, Faculty of Medicine, University of Oslo, Norway; 4 Rikshospitalet University Hospital, 0027 Oslo, Norway

## Abstract

**Background:**

Patients with knee osteoarthritis [OA] are commonly treated by physiotherapists in primary care. Measuring physiotherapy performance is important before developing strategies to improve quality. The purpose of this study was to measure physiotherapy performance in patients with knee OA by comparing clinical practice to evidence from systematic reviews.

**Methods:**

We developed a data-collection form and invited all private practitioners in Norway [n = 2798] to prospectively collect data on the management of one patient with knee OA through 12 treatment session. Actual practice was compared to findings from an overview of systematic reviews summarising the effect of physiotherapy interventions for knee OA.

**Results:**

A total of 297 physiotherapists reported their management for patients with knee OA. Exercise was the most common treatment used, provided by 98% of the physiotherapists. There is evidence of high quality that exercise reduces pain and improves function in patients with knee OA. Thirty-five percent of physiotherapists used acupuncture, low-level laser therapy or transcutaneous electrical nerve stimulation. There is evidence of moderate quality that these treatments reduce pain in knee OA. Patient education, supported by moderate quality evidence for improving psychological outcomes, was provided by 68%. Physiotherapists used a median of four different treatment modalities for each patient. They offered many treatment modalities based on evidence of low quality or without evidence from systematic reviews, e.g. traction and mobilisation, massage and stretching.

**Conclusion:**

Exercise was used in almost all treatment sessions in the management of knee OA. This practice is desirable since it is supported by high quality evidence. Physiotherapists also provide several other treatment modalities based on evidence of moderate or low quality, or no evidence from systematic reviews. Ways to promote high quality evidence into physiotherapy practice should be identified and evaluated.

## Background

Osteoarthritis [OA] is the most common condition affecting synovial joints [1]. The number of persons affected by OA in the western world will increase because its prevalence increases with age [1]. Patients with knee OA are managed in primary care, and they represent a large group seen by physiotherapists. An overview of systematic reviews covering physiotherapy interventions for patients with osteoarthritis of the knee demonstrates that exercise can reduce pain and improve function in patients with knee OA [2]. It also indicates that low-level laser, transcutaneous electrical nerve stimulation and acupuncture can reduce pain, and that psychoeducation, including patient education and self-management programmes, can improve psychological outcomes. Thus, physiotherapy can improve pain and function and play an important role in the management of patients with knee OA.

Improving the quality of care is a major issue for all health care systems, and measuring performance is essential for the planning and evaluation of quality improvement strategies [3-5]. Measuring performance means comparing actual clinical practice to desired clinical practice. Patient perspectives of care and patient outcomes can also be included in performance measurements [3]. Most performance studies in physiotherapy have described management of low back pain [6-9]. Although OA is a highly prevalent disease, little is known about the performance, including physiotherapy for patients with OA [10].

The aim of this study was to measure physiotherapy performance in patients with knee OA by comparing actual clinical practice to evidence from systematic reviews.

## Methods

The study was conducted among private physiotherapy practitioners in Norway, who are integrated into primary health care. The National Committees for Research Ethics in Norway approved the protocol for the study.

### Data collection form

For the purpose of this study, we developed a paper-based data-collection form to register actual clinical practice for patients with knee OA. We started the development by visiting several practices, observing physiotherapists treating patients with knee OA. In two one-day meetings, ten clinicians invited through The Norwegian Physiotherapy Association developed, piloted and revised the data-collection form in collaboration with the researchers. We piloted the form among 10 physiotherapists and assessed the reliability of the form using 15 independent observations of treatment sessions. We evaluated the relationship between data entered independently by the observer, who was an experienced physiotherapist, and data entered by the treating physiotherapists and calculated kappa scores.

The final form was in three parts (see variables and the original data-collection form in Additional files 1 &2). Part one covered patient characteristics, the physiotherapy examination and the treatments goals, e.g. the patients gender and age, time since diagnosis, type of pain classified in six categories (e.g., pain at night, rest, weight bearing, start of activity) and intensity of pain measured on a ten point visual analog scale (VAS), co-morbidity and the physiotherapist's judgement of physical activity level and patient weight. The aims of treatment, e.g. reducing pain, improving function, muscle strength, aerobic capacity or increasing knowledge were assessed on a six point scale from "Not important at all" to "Very important".

Part two was designed to report the treatment modalities used in each session during 12 sessions. This part contained a list of 35 different treatments, e.g. types of exercise, massage, traction, hot packs, physical modalities, information and patient education. We also collected information about whether the patients were treated individually or in groups. We chose 12 treatment sessions because this is the number most often used when general practitioners refer patients to physiotherapy in Norway. Part three collected information on characteristics of the physiotherapists, e.g. gender, age, years since qualification, work setting and postgraduate education. A designer contributed to the lay-out to create a user-friendly form.

### Recruitment

We invited all private practitioners, identified by membership of The Norwegian Physiotherapy Association in February 2006, to participate in the study [n = 2798]. We asked the physiotherapists to report the management of the first patient with knee OA referred to their practice [one case], and to complete the form at every treatment session. The diagnosis should be confirmed by x-ray or magnetic resonance imaging. Patient who had a knee arthrosplasty or postoperative treatments were excluded.

In response to an invitation letter sent out in May 2006, 744 physiotherapists replied that they did not normally treat patients with OA, or that they had other reasons for not being eligible. In August 2006 we distributed the anonymous data-collection form with a pre-paid return envelope to the remaining physiotherapists [n = 2054]. To increase the response rate, we sent two reminder letters to all, and one e-mail postcard to those with an e-mail address, and we contacted practices with more than five physiotherapists by telephone. The study was also described in the *Norwegian Physiotherapy Journal *and in a newsletter sent to all private practitioners. The data collection period was nine months, from August 2006 to May 2007.

### Research evidence and performance

We have previously summarised the evidence from systematic reviews on physiotherapy interventions for patients with knee OA in an overview, and assessed the quality of evidence for each intervention, comparison and outcome [2]. The quality of evidence for the interventions was categorised as high, moderate or low, or as no evidence from systematic reviews. The quality of the evidence indicates the extent to which one can be confident that the estimate of effect is correct. High quality evidence indicates that further research is very unlikely to change our confidence in the estimate of effect.

We measured physiotherapy performance by comparing practice reported in the data-collection forms to the findings from the overview. If the physiotherapist used interventions that were supported by evidence for improving patient outcomes of high or moderate quality, we interpreted the practice as desirable. Even though there was a lack of evidence for the effects of giving advice, we considered giving advice and information about physical activity and weight reduction as desirable practice.

### Analysis

We performed descriptive analysis, based on frequency distribution and percentages, to assess characteristics of the patients and the physiotherapists, and the treatments used. Different types of exercise, e.g. exercises aimed to increase muscle strength, aerobic capacity, coordination or range of motion, were merged into one treatment modality. We classified the use of each treatment modality into three categories, "not used", "used in up to 80% of the sessions" and "used in more than 80% of the sessions". "Used in more than 80% of the sessions" was interpreted as treatment used in almost all sessions. We also calculated the total number of different treatment modalities used by each physiotherapist through the sessions.

## Results

We received a response from 527 therapists. Among these, 297 had treated one patient with knee OA and had completed the data-collection form. The responders that did not complete the form (n = 230) reported various reasons for not completing, e.g. no patient referred during the study period (n = 109), not working in clinical practice (n = 41) or specialist in other areas such as neurology, child or mental health (n = 46).

When assessing the reliability of the data-collection form we found that the different items had a kappa score that varied from 0.8 to 1.0. For some types of exercise, e.g exercise aimed at increasing strength, coordination and stability, the score was lower.

The mean age of the physiotherapists was 47 years [SD = 11]. Almost half [47%] were women [Table 1]. Patients had a mean age of 65 years [SD = 11], and 67% were women. Pain intensity during the last week was 5.9 [SD = 2.1] on a 10-point visual analogue scale [VAS]. Almost half of the patients [46%] suffered from pain during night, or at rest. More than half had bilateral knee OA, and 32% were diagnosed more than five years ago. Thirty three percent were considered overweight, and 31% had important co-morbidity, most frequently reported was cardiovascular diseases or low back pain. Fifty percent of the patients were referred to physiotherapy for knee OA for the first time.

**Table 1:** Characteristics of physiotherapists (N = 297)

Variable	
Age [mean [SD]]	47 [11]
Year since qualification [mean [SD]]	21 [12]
Women [%]	47
Practice setting [%]	
Single practice	15
2–5 colleagues	58
More than 5 colleagues	27
Postgraduate education [%]	26
Specialist [%]	11
Masters degree [%]	3

The most important aim for the treatment, as reported by the therapists, was to reduce pain [92%], followed by increasing muscle strength [85%].

Exercise was used by all but six physiotherapists [2%], and 86% used exercise in almost all sessions; 11% of physiotherapists provided exercise as the only treatment at all 12 sessions. As described in Table 2, there is high quality evidence that exercise reduces pain and improves physical function in patients with knee OA. Type of exercise, e.g., improving muscle strength, gait, range of motion and stability varied widely, both within and across sessions. Muscle strengthening exercises were most commonly used (90%). Few physiotherapists (17%) treated their patients in a group setting.

**Table 2:** Number [%] of treatment modalities used in the management of patients with knee osteoarthritis according to quality of evidence from systematic reviews (SR)

**Type of intervention**	**Not used at all**	**Used in up to 80% of the sessions**	**Used in more than 80% of the sessions**	**Quality of evidence**
Exercise	6 [2]	35 [12]	256 [86]	High for pain reduction and improved physical function Moderate for no improvement in psychological outcomes
TENS (transcutaneous electrical nerve stimulation)	260 [88]	16 [5]	21 [7]	Moderate for pain reduction
Low level laser therapy	265 [89]	22 [7]	10 [4]	Moderate for pain reduction
Acupuncture (manual, electrical and trigger point)	237 [80]	40 [14]	20 [7]	Moderate for pain reduction
Short wave therapy (and pulsed electromagnetic energy)	268 [90]	16 [5]	13 [4]	Moderate for no reduction in pain or improvement in physical function
Patient education, self-management and psychoeducation	53 [18]	214 [72]	29 [10]	Moderate for improving psychological outcomes Moderate for no difference in pain or physical function
Ultrasound	249 [84]	21 [7]	27 [9]	Low for all outcomes
Thermotherapy (heat packs)	251 [85]	20 [7]	26 [8]	Low for all outcomes
Thermotherapy (cold packs)	278 [94]	12 [4]	7 [2]	Low for all outcomes
Braces and orthosis	273 [92]	21 [7]	3 [1]	Low for all outcomes
Tape	286 [96]	10 [3]	1 [0]	No evidence from SR
Massage	137 [46]	69 [24]	91 [30]	No evidence from SR
Traction	158 [53]	60 [20]	78 [26]	No evidence from SR
Stretching	158 [53]	57 [19]	81 [27]	No evidence from SR
Advice about physical activity	32 [11]	220 [74]	45 [15]	No evidence from SR
Advice about weight reduction among 102 patients considered overweight	43 [42]	55 [54]	4 [4]	No evidence from SR

There is evidence of moderate quality that transcutaneous electrical nerve stimulation [TENS], low-level laser therapy and acupuncture reduce pain. Each of these modalities were used by less than 25% of the therapists [Table 2]. Moderate quality evidence suggests that short-wave or pulsed electromagnetic energy has no effect on outcomes for knee OA. This modality was provided by only 10% of physiotherapists.

The physiotherapists applied a median number of four [range 1–10] different treatment modalities for each patient throughout the sessions. Massage, traction/mobilisation and stretching were the next most common modalities after exercise, and were applied in approximately half of patients [Table 2]. There is no evidence from systematic reviews about the effect of these treatments.

There is evidence of moderate quality that psychoeducation, including patient education and self-management programmes improve psychological outcomes, e.g., scales of psychological disability, mental functioning, self-efficacy or depressive symptoms. Sixty eight percent used interventions that were classified as psychoeducation, such as education about OA and coping with the disease. Almost all physiotherapists [90%] provided information and guidance about physical activity, and 76% prescribed a home exercise programme.

The physiotherapists provided advice and information about weight reduction to 59 [58%] of the 102 patients that they considered overweight. On the other hand, almost all patients that the physiotherapist assessed to need more physical activity [n = 101] received advice and support for increasing activity level [n = 92].

## Discussion

To our knowledge, this is the first study of physiotherapy performance for patients with knee OA. The study describes clinical practice in terms of individual patients, as recorded prospectively by therapists during every treatment session. We compared the treatment to findings from an overview of systematic reviews. Quality of care includes many elements. We have studied one important factor that contributes to quality, – the factor of clinical effectiveness.

Almost all therapists in this study used exercise in all treatment sessions. This current practice is desirable, since it is supported by evidence of high quality. Less than 35% of physiotherapists used acupuncture, low-level laser therapy or TENS which have moderate-quality evidence for reducing pain. In addition, physiotherapists used many treatment modalities with low-quality evidence or no evidence from systematic reviews, e.g., traction, massage and stretching.

The physiotherapists provided different types of exercises. Because there is no evidence from systematic reviews to support one specific type or dose [11], we merged different types of exercise into one treatment modality. Clearly, we lost some information about practice by this procedure, but as long as no type of exercise is shown to be more beneficial than another we think this was reasonable. We also categorised different information modalities, but separated simple information about exercise and weight reduction from pscychoeducation and self-management programmes. There is clearly an overlap between these interventions that might introduce information bias or misclassification in this study. The effects of advice and information about exercise and weight reduction provided by physiotherapists to patients with knee OA is unclear, although systematic reviews have demonstrated that exercise and weight reduction improve outcomes in knee OA [2]. However, professional advice and guidance with continued support can encourage people from the general population to be more physically active [12]. Long-term adherence to exercise is required to maintain the benefits of exercise in knee OA, and because long-term adherence requires regular motivation, supervision and monitoring [12], physiotherapists should include such guidance in all treatment sessions. Although many gave advice about physical activity, few physiotherapists [15%] reported having provided this in more than 80% of the sessions.

Only 58% of the patients that the physiotherapists categorized as overweight were given information and advice about weight reduction. The therapists rated subjectively if the patient was overweight. This method might be prone to bias because we do not know how this measure compares with body mass index, which is commonly used to identify overweight. However, clinical judgement and experience might be as important as body mass index for offering patients advice about weight reduction. There are many plausible explanations why many physiotherapists did not focus on weight reduction, e.g., they do not have enough knowledge and/or skills on how to address the problem, the topic is too intimate or they provide advice on physical activity instead. Still we think that physiotherapists might contribute to the positive outcomes of weight reduction by supervision and guidance, perhaps in cooperation with a dietician.

Our findings are comparable to studies of physiotherapy performance for low back pain which demonstrate that adherence to guidelines varies across different treatment modalities [6,9,13]. Treatments for which evidence is limited or absent are also frequently used [6,9]. Interestingly, our study shows that electrotherapy modalities that can reduce pain supported by moderate quality evidence were used by less than 35% of the physiotherapists. In studies of low back pain [6,14], electrotherapy was more frequently used even though there was no evidence of effect. However, interventions should always be specified to meet the need from individual patients, and the physiotherapists might choose not to use these modalities if the patient had mild pain. If providing electrotherapy, the physiotherapist should choose modalities supported by moderate quality evidence instead of modalities with no evidence, or with evidence of no effect. Still, almost all therapists used exercise, and exercise can also reduce pain. Though, we can not argue that the therapists were providing inadequate care by not using low level laser, TENS or acupuncture.

There are some limitations to this study. The response rate was low, and this might be a threat to the validity of the data of physiotherapy performance because the therapists that responded might have different practice pattern than the study population. We feared that a low response rate might be a problem, and we tried to develop a strategy to get a large and unbiased sample of responses from Norwegian physiotherapists. We invited all private practitioners in Norway to the study. We used finding from a systematic review on how to increase response rate [15]. We contacted the physiotherapists before the study started, the data-collection form was user-friendly with pre-paid return and we had several follow-up contacts. In addition, we enclosed a bar of dark chocolate with a sticker saying "Thank you for contributing to physiotherapy documentation" randomly to half of the physiotherapists.

The physiotherapists who participated were comparable to private practitioners in Norway regarding age [mean age reported by the Norwegian Physiotherapists Association is 48], although a higher proportion of men responded to our study. We have no additional information about the non-responding physiotherapists. Surprisingly many physiotherapists reported that they did not treat a patient with knee OA during the study period. This might also be the case for many of the non-responders.

Other studies of physiotherapy performance in primary care that have used a prospective design have experienced the same lack of participation [6,16]. When Swinkels et al established a network to collect practice data on a continuous basis in The Netherlands they only collected data from 90 physiotherapists [9].

Another potential source of bias is the self-selection of patients, because the therapists might choose patients that are not representative to patients normally treated in private practice. We asked the physiotherapists to report the management of the first patient with knee OA. The characteristics of the patients in the study are comparable to patients included in 36 trials in a systematic review on physical interventions for patients with OA [17]. The mean age was 65.1 years and the mean baseline pain score was 62.9 on a 100-mm VAS.

We collected data by self-report from the therapist. Self-report of practice might represent a threat to validity because some therapists might report treatments that they do not perform. Some might also adopt new practice pattern because they think it is expected. This might mean that self-reported adherence rates to guidelines could exceed the rate measured by medical records or observation [18]. There might be variation in how the therapists interpret and respond to the data collection from.

We measured performance by comparing practice to findings from systematic reviews. For some interventions we lack evidence because we did not identify any systematic reviews. Evidence of high quality from primary studies not included in systematic reviews might be available for such interventions. This is clearly a limitation to our approach. Secondly, some reviews needed updating. Inclusion of new primary studies might change the estimates of effect and the quality of evidence. Finally, it is crucial to remember that "no evidence from systematic reviews" does not imply "evidence of no effect".

It is difficult to measure physiotherapy performance because physiotherapy practice is complex. Treatment can differ both within and across sessions. Type, dose and frequency vary and the interaction and communication between patient and therapist are important parts of the therapy. In the present study we assessed performance for one measurable part of physiotherapy practice, but we excluded interpersonal communication, structural aspects of care, organisational culture, teamwork and access. These are other important parts of high quality physiotherapy care. Multiple data collection methods might be used to get a more comprehensive picture of actual physiotherapy practice.

Despite clear limitations in our methods, this study contributes to the knowledge about physiotherapy performance in patients with knee OA. We need research to develop valid and reliable methods to measure physiotherapy performance in primary care, as well as research on how to bridge research and clinical practice. Specifically, we should identify effective ways to promote interventions supported by high quality evidence. Finally, in order to be able to measure performance in physiotherapy, we need more research and more systematic reviews on the effects of physiotherapy interventions for patients with knee OA. Because physiotherapists use exercise regularly for patients with knee OA, and there are different opinions about optimal exercise regimen, studies should compare different types, settings, intensities and volumes of exercise. Interventions that are frequently used by physiotherapists without evidence from systematic reviews, e.g., traction, massage and stretching for patients with knee OA, should be tested in rigorous trials and summarised in reviews.

## Conclusion

This study provides information about physiotherapy performance in patients with knee OA. Exercise is the most common treatment and this is supported by high quality evidence. Physiotherapists also provide several treatment modalities based on moderate and low quality evidence of benefit, or without evidence from systematic reviews. We need more research to develop and identify the best methods to measure physiotherapy performance in primary care.

## Abbreviations

OA: osteoarthritis; SD: standard deviation: TENS: transcutaneous electrical nerve stimulation; VAS: visual analogue scale.

## Competing interests

The authors declare that they have no competing interests.

## Authors' contributions

GJ wrote the protocol and designed the study, developed the data-collecting instrument, performed the analysis and drafted the first version of the manuscript. KTD contributed to designing the study, piloted the instrument, entered data into SPSS and revised drafts of the manuscript. IH and SF contributed to the idea of the project and to design and analysis and revised drafts of the manuscript. All authors approved the final manuscript

## Pre-publication history

The pre-publication history for this paper can be accessed here:



## Supplementary Material

Additional file 1 Variable list. Click here for file

Additional file 2 Data collection form. Click here for file
