# Supplementary material for: Measuring physiotherapy performance in patients with osteoarthritis of the knee: A prospective study
Source: BMC Health Serv Res. 2008 Jul 8;8:145. doi: 10.1186/1472-6963-8-145 (PMC2475531; doi:10.1186/1472-6963-8-145)
Supplement: Additional file 1 — Variable list. [file 1472-6963-8-145-S1.doc]

| **Variables** | **Response categories** |
| --- | --- |
| *Patient characteristics* |  |
| Gender | Male; Female |
| Age | Year of birth |
| Work status | Employed; On sick leave; Disability pension/early retirement; Home maker; Retired; Other |
| Time since diagnosis | Less than one year; 1-5 years; More than 5 years |
| Pervious knee injury | Yes; No; Don’t know |
| Pervious knee surgery | Yes; No; If yes, what and when? |
| Bilateral knee OA | Yes; No; Don’t know |
| Type of pain | Pain during activity; Pain during weight load; Pain at rest; Pain at night; Pain at start of movement; Not assessed |
| Intensity of pain | VAS 0 - 10 |
| Varus/valgus | Yes; No; Don’t know; Not assessed |
| Hydrops | Yes; No; Don’t know; Not assessed |
| Medication | NSAIDS; Paracet, Glucosamine; Other; Doesn’t use |
| Co-morbidity | Yes; No;  If yes, what type of co-morbidity? |
| Overweight (subjectively assessed by the physiotherapists) | Yes; No; Don’t know; Not assessed |
| Need more physical activity (subjectively assessed by the physiotherapists) | Yes; No; Don’t know; Not assessed |
| Previous physiotherapy for the same condition | No, first time; Yes, once; Yes, more than once |
| *Assessment* |  |
| Physiotherapist used outcome measures? | Yes; No;  If yes, which measures were used |
| Findings | 6 predefined findings on different ICF-levels (impairment, function and disability) reported as:  Yes; No; Not assessed |
| Treatment goals were defined | Yes; No |
| Patients treatment goals were clarified | Yes; No |
| Who defined the treatment goals | The therapist; The patient; Shared |
| Treatment goals | 13 predefined goals on different ICF-levels (impairment, function and disability) reported on a 6 point scale for each goal from “Not important at all” to “Very important” |
| Follow-up goals defined | Yes; No |
| *Setting and treatment modality used in each session through 12 treatment sessions* |  |
| Setting of treatment | Individual treatment; Individual training with some supervision; Group (specific exercise); Group (general exercise) |
| Exercise for:  Pain reduction  Muscle strengthening  Range of motion  Stability  Coordination  Aerobic capacity  Relaxation  ADL-activities | Each type of exercise reported at every treatment session through 12 sessions as:  Used; Not used |
| All other relevant treatment modalities and types of advise and patient education  (20 modalities and 10 types of advise/education listed) | Each type of treatment modality reported at every treatment session through 12 sessions as:  Used; Not used |
| *Variables reported at end of 12 treatment sessions* |  |
| Changed the treatment goals during the treatment period? | Yes; No |
| Evaluated the goals by end of treatment period? | Yes; No |
| Goals reached at end of treatment period? (assessed by physiotherapists by end of 12 sessions) | Yes; No; Partially |
| Will the patient continue physiotherapy treatment after the 12 sessions reported? | Yes, will continue; Will continue unsupervised exercise; No, will not continue |
| *Physiotherapists characteristics* |  |
| Gender | Male; Female |
| Age | Year of birth |
| Year since qualification | Year |
| Postgraduate and continuing education | Continuing education; Postgraduate education; Master Degree; Specialists in area of physiotherapy |
| Type of practice | Solo practice; 2-5 colleagues; More than 5 colleagues |
| Operating Contribution | Yes; No;  If yes, list percent contribution |
| Internet access at work | Yes; No |
| Exercise facility in separate room | Yes; No |
| Use of electronically journal system | Yes, all of us; Yes, some; None |
| Number articles read last 6 months | None; Less than 5; 5-10; More than 10 |
| Used medical databases last 6 months | Yes; No;  If yes, list database: |
| Problems finding relevant information | Often; From time to time; Rarely; Never |
| Read article about knee OA the past year | Yes; No |
| Attended a lecture or course on knee OA last two years | Yes; No |
