# Supplementary material for: Measuring physiotherapy performance in patients with osteoarthritis of the knee: A prospective study
Source: BMC Health Serv Res. 2008 Jul 8;8:145. doi: 10.1186/1472-6963-8-145 (PMC2475531; doi:10.1186/1472-6963-8-145)
Supplement: Additional file 2 — Data collection form. [file 1472-6963-8-145-S2.pdf]

Nasjonalt kunnskapssenter for helsetjenesten
Postboks 7004, St. Olav plass, N-0130 Oslo
Tlf: (+47) 23 25 50 00 Faks: (+47) 23 25 50 10
www.kunnskapssenteret.no

ID

Fysioterapi
praksis

FYSIOTERAPI TIL PASIENTER MED KNELEDDSARTROSE
Kartlegging av praksis

Skjemaet er tredelt:

Del 1 skal gi informasjon om pasienten og mål for behandlingen. Det skal fylles ut etter første eller annen gangs konsultasjon og vil ta ca 5 min

Del 2 skal kartlegge behandlingstiltak, skal fylles ut under behandlingsforløpet og vil ta ca 1/2 min per gang

Del 3 skal gi informasjon om behandlende fysioterapeut, skal fylles ut en gang og vil ta ca 5 min

DEL 1

INFORMASJON OM PASIENTEN MED KNELEDDSARTROSE
FUNN OG MÅL FOR BEHANDLING

Mann	Kvinne	Fødselsår
♂ ☐	♀ ☐	☐☐☐☐

Arbeidssituasjon

☐ I arbeid ☐ Sykmeldt ☐ Uføretrygdet ☐ Hjemmeværende ☐ Pensjonist ☐ Annet

---

Hvor lenge er det siden diagnose ble stilt?

☐ Under 1 år ☐ 1-5 år ☐ Over 5 år

Har pasienten tidligere skadet kneet, for eksempel menisk eller korsbånd?

☐ ja ☐ nei ☐ vet ikke

Er pasienten operert i kneet ?

☐ ja ☐ nei Hvis ja, hva slags operasjon og når?

Har pasienten artrose i begge knær ?

☐ ja ☐ nei ☐ vet ikke

Type smerter?

☐ Smerter v/ spesielle aktiviteter ☐ Belastningssmerter ☐ Startsmerter
☐ Hvilesmerter ☐ Nattsmerter ☐ Ikke kartlagt

Angi pasientens smerter (siste uke) på skalaen hvor 0 er ingen smerter, og 10 er verst tenkelige smerter (sett et kryss i aktuell rute)

0 ☐☐☐☐☐☐☐☐☐☐☐ 10

Har pasienten forøket valgus eller varusstilling?

☐ ja ☐ nei ☐ vet ikke ☐ Ikke vurdert

Har pasienten hydrops/hevelse i kneet?

☐ ja ☐ nei ☐ vet ikke ☐ Ikke vurdert

Bruker pasienten medikamenter for kneartrosen?

☐ NSAIDS ☐ Paracet ☐ Glucosaminer ☐ Annet ☐ Bruker ikke

Har pasienten andre sykdommer eller helseplager av betydning for behandlingen?

☐ ja ☐ nei Hvis ja, hvilke sykdommer eller plager:

Vurderer du pasienten som overvektig?

☐ ja ☐ nei ☐ vet ikke ☐ Ikke vurdert

Vurderer du pasienten til å være for lite fysisk aktiv?

☐ ja ☐ nei ☐ vet ikke ☐ Ikke vurdert

Har pasienten fått fysioterapibehandling for kneleddsartrose før?

☐ Ja, flere ganger ☐ Ja, en gang tidligere ☐ Nei

Benyttet du funksjonsskåreskjema eller smerterskala i undersøkelsen?

(for eksempel WOMAC, COOP WONCA eller VAS-skala)

☐ ja ☐ nei

Hvis ja, – hvilke?

Ved undersøkelsen, identifiserte du funn relatert til:

	**JA**	**NEI**	**IKKE VURDERT**
Smerter	☐	☐	☐
Bevegelighet (fleksjon/ekstensjon)	☐	☐	☐
Bevegelighet (patellas bevegelighet)	☐	☐	☐
Muskulatur (styrke el utholdenhet)	☐	☐	☐
Koordinasjon	☐	☐	☐
Stabilitet	☐	☐	☐
Gange	☐	☐	☐
ADL-funksjon (dagligliv el fritid)	☐	☐	☐
Annet:			

Ble det satt konkrete mål for behandlingen?

☐ ja ☐ nei

Ble pasientens mål klarlagt?

☐ ja ☐ nei

Hvis det ble satt mål, hvem satte de endelige målene?

☐ Pasienten ☐ Fysioterapeuten ☐ I samarbeid

Mål for behandlingen (ranger viktighet på en skala fra 0 – 5 ved å sette kryss i aktuelle ruter)

	IKKE VIKTIG 0	1	2	3	4	5 SVÆRT VIKTIG
Redusere smerter	☐	☐	☐	☐	☐	☐
Bedre bevegelighet	☐	☐	☐	☐	☐	☐
Bedre koordinasjon	☐	☐	☐	☐	☐	☐
Bedre stabilitet	☐	☐	☐	☐	☐	☐
Bedre muskelstyrke/utholdenhet	☐	☐	☐	☐	☐	☐
Bedre avspenning	☐	☐	☐	☐	☐	☐
Bedre ADL-funksjon	☐	☐	☐	☐	☐	☐
Bedre gangfunksjon	☐	☐	☐	☐	☐	☐
Bedre sosial fungering	☐	☐	☐	☐	☐	☐
Øke kunnskap hos pasienten	☐	☐	☐	☐	☐	☐
Påvirke fysisk aktivitetsnivå	☐	☐	☐	☐	☐	☐
Redusere vekt	☐	☐	☐	☐	☐	☐
Annet	☐	☐	☐	☐	☐	☐

Ble det satt noen langsiktige mål? (sett opp i prioritert rekkefølge dersom flere mål)

☐ ja ☐ nei

Mål 1: Mål 2:

DEL 2

BEHANDLINGSTILTAK

Sett kryss for hver behandling

**BEHANDLING NR**	**1**	**2**	**3**	**4**	**5**	**6**	**7**	**8**	**9**	**10**	**11**	**12**	**Sum**
**DATO**													
**VARIGHET AV HVER BEHANDLING** (ca i minutter)													
Behandling på benk	☐	☐	☐	☐	☐	☐	☐	☐	☐	☐	☐	☐	
Individuell behandling/trening	☐	☐	☐	☐	☐	☐	☐	☐	☐	☐	☐	☐	
Gruppebehandling/trening med spesifikke øvelser	☐	☐	☐	☐	☐	☐	☐	☐	☐	☐	☐	☐	
Egentrening med spesifikke øvelser	☐	☐	☐	☐	☐	☐	☐	☐	☐	☐	☐	☐	
Gruppetrening med generelt program	☐	☐	☐	☐	☐	☐	☐	☐	☐	☐	☐	☐	
**ØVELSER med mål å påvirke:**													
Muskelstyrke	☐	☐	☐	☐	☐	☐	☐	☐	☐	☐	☐	☐	
Bevegelighet	☐	☐	☐	☐	☐	☐	☐	☐	☐	☐	☐	☐	
Smerter	☐	☐	☐	☐	☐	☐	☐	☐	☐	☐	☐	☐	
Koordinasjon	☐	☐	☐	☐	☐	☐	☐	☐	☐	☐	☐	☐	
Kondisjon	☐	☐	☐	☐	☐	☐	☐	☐	☐	☐	☐	☐	
Stabilitet	☐	☐	☐	☐	☐	☐	☐	☐	☐	☐	☐	☐	
Avspenning	☐	☐	☐	☐	☐	☐	☐	☐	☐	☐	☐	☐	
ADL-funksjon	☐	☐	☐	☐	☐	☐	☐	☐	☐	☐	☐	☐	
Annet, spesifiser:	☐	☐	☐	☐	☐	☐	☐	☐	☐	☐	☐	☐	
**ANDRE BEHANDLINGSTILTAK**													
Ergometersykkel	☐	☐	☐	☐	☐	☐	☐	☐	☐	☐	☐	☐	
Bassengtrening	☐	☐	☐	☐	☐	☐	☐	☐	☐	☐	☐	☐	
Massasje/bløtdelsbehandling	☐	☐	☐	☐	☐	☐	☐	☐	☐	☐	☐	☐	
Leddmobilisering/traksjon	☐	☐	☐	☐	☐	☐	☐	☐	☐	☐	☐	☐	
Tøyning	☐	☐	☐	☐	☐	☐	☐	☐	☐	☐	☐	☐	
Varme	☐	☐	☐	☐	☐	☐	☐	☐	☐	☐	☐	☐	
Is	☐	☐	☐	☐	☐	☐	☐	☐	☐	☐	☐	☐	
Ultralyd	☐	☐	☐	☐	☐	☐	☐	☐	☐	☐	☐	☐	
TENS	☐	☐	☐	☐	☐	☐	☐	☐	☐	☐	☐	☐	
Laser	☐	☐	☐	☐	☐	☐	☐	☐	☐	☐	☐	☐	
Kortbølge	☐	☐	☐	☐	☐	☐	☐	☐	☐	☐	☐	☐	
Sjokkbølge	☐	☐	☐	☐	☐	☐	☐	☐	☐	☐	☐	☐	
Annen elektroterapi	☐	☐	☐	☐	☐	☐	☐	☐	☐	☐	☐	☐	

Dersom tiltakene ultralyd, TENS, laser, kortbølge, sjokkbølge eller annen elektroterapi er gitt, oppgi intensitet og varighet per gang: ______ (intensitet) ____ minutter

BEHANDLING NR	1	2	3	4	5	6	7	8	9	10	11	12	Sum
Tape	☐	☐	☐	☐	☐	☐	☐	☐	☐	☐	☐	☐	
Ortose/såler	☐	☐	☐	☐	☐	☐	☐	☐	☐	☐	☐	☐	
Slyngeterapi	☐	☐	☐	☐	☐	☐	☐	☐	☐	☐	☐	☐	
Manuell akupunktur	☐	☐	☐	☐	☐	☐	☐	☐	☐	☐	☐	☐	
Elektrisk akupunktur	☐	☐	☐	☐	☐	☐	☐	☐	☐	☐	☐	☐	
Triggerpunktbehandling	☐	☐	☐	☐	☐	☐	☐	☐	☐	☐	☐	☐	

INFORMASJON OG RÅDGIVNING													
Informasjon om fysisk aktivitet og mosjon	☐	☐	☐	☐	☐	☐	☐	☐	☐	☐	☐	☐	
Råd og tilrettelegging for egentrening og mosjon	☐	☐	☐	☐	☐	☐	☐	☐	☐	☐	☐	☐	
Hjemmeprogram med spesifikke øvelser	☐	☐	☐	☐	☐	☐	☐	☐	☐	☐	☐	☐	
Informasjon og råd om vektreduksjon	☐	☐	☐	☐	☐	☐	☐	☐	☐	☐	☐	☐	
Informasjon om leddvern	☐	☐	☐	☐	☐	☐	☐	☐	☐	☐	☐	☐	
Råd om ro og avlastning	☐	☐	☐	☐	☐	☐	☐	☐	☐	☐	☐	☐	
Samtale om å leve med kneartrose	☐	☐	☐	☐	☐	☐	☐	☐	☐	☐	☐	☐	
Hjelpemidler	☐	☐	☐	☐	☐	☐	☐	☐	☐	☐	☐	☐	
Tilrettelegging i hjemmet eller på arbeidsplass	☐	☐	☐	☐	☐	☐	☐	☐	☐	☐	☐	☐	
Andre, spesifiser:													

Kommentarer til behandlingsforløpet m.m.

EVALUERING AV MÅL (fylles ut ved slutten av behandlingsserien)

Er målene evaluert underveis i behandlingen ?

☐ ja ☐ nei

Er målene evaluert etter fullført behandling?

☐ ja ☐ nei

Er målene oppnådd?

☐ ja ☐ nei ☐ delvis

Skal pasienten ha flere behandlinger utover denne serien eller fortsette med egentrening?

☐ skal forsette med behandling ☐ skal forsette med egen trening ☐ skal ikke forsette

KOMMENTARER

DEL 3

INFORMASJON OM BEHANDLENDE FYSIOTERAPEUT

(fylles ut en gang)

Mann	Kvinne	Fødselsår
♂ ☐	♀ ☐	☐☐☐☐

År siden avsluttet grunnutdanning i fysioterapi: ☐☐

Etter- og videreutdanning

☐ Etterutdanningskurs (korte kurs) siste 3 år
☐ Spesialist i fysioterapi
☐ Videreutdanning
☐ Hovedfag/mastergrad

Oppgi type spesialitet, videreutdanning og/eller grad:

Praksistype

☐ Jobber alene ☐ 2-5 sammen på instituttet ☐ Mer enn 5 sammen

% stilling: ☐☐☐

Driftstilskudd

☐ ja ☐ nei Oppgi % andel: ☐☐☐

Tilgang til Internett på jobben

☐ ja ☐ nei

Tilgang til treningssal på instituttet

☐ ja ☐ nei

Benytter elektronisk journal

☐ Ja, alle på instituttet ☐ Noen av oss ☐ Ingen

Hvor mange fagartikler har du lest siste 6 måneder?

☐ Ingen ☐ Under 5 ☐ 5-10 ☐ Over 10

Har du i løpet av siste 6 måneder benyttet elektroniske databaser for å søke svar på faglige spørsmål?

☐ ja ☐ nei

Hvis ja, – hvilke?

Hvis du tar hele din arbeidssituasjon i betraktning, har du problemer med å få tak i relevant informasjon i faglige spørsmål?

☐ ofte ☐ av og til ☐ sjelden ☐ aldri

Har du lest noen fagartikkel om kneleddsartrose siste år?

☐ ja ☐ nei

Har du hørt foredrag eller deltatt på kurs om kneleddsartrose siste to år?

☐ ja ☐ nei

KONTAKT

Har du spørsmål, kontakt

Gro Jamtvedt
Forsker og fysioterapeut
T: 97 51 81 44
E: grj@kunnskapssenteret.no

Kristin Thuve Dahm
Forskningsassistent og fysioterapeut
T: 46 40 04 52
E: ktd@kunnskapssenteret.no

Nasjonalt kunnskapssenter for helstjenesten
Postboks 7004, St. Olavs plass, N–0130 Oslo
